# Racial variations of adverse perinatal outcomes: A population-based retrospective cohort study in Ontario, Canada

**DOI:** 10.1371/journal.pone.0269158

**Published:** 2022-06-30

**Authors:** Qun Miao, Yanfang Guo, Erica Erwin, Fayza Sharif, Meron Berhe, Shi Wu Wen, Mark Walker

**Affiliations:** 1 Better Outcomes Registry & Network Ontario, Ottawa, Ontario, Canada; 2 School of Epidemiology and Public Health, University of Ottawa, Ottawa, Ontario, Canada; 3 Children’s Hospital of Eastern Ontario Research Institute, Ottawa, Ontario, Canada; 4 OMNI Research Group, Ottawa Hospital Research Institute, Ottawa, ON, Canada; 5 Department of Obstetrics and Gynecology, University of Ottawa, Faculty of Medicine, Ottawa, Ontario, Canada; Tulane University School of Public Health and Tropical Medicine, UNITED STATES

## Abstract

**Introduction:**

Racial differences in adverse maternal and birth outcomes have been studied in other countries, however, there are few studies specific to the Canadian population. In this study, we sought to examine the inequities in adverse perinatal outcomes between Black and White pregnant people in Ontario, Canada.

**Methods:**

We conducted a population-based retrospective cohort study that included all Black and White pregnant people who attended prenatal screening and had a singleton birth in any Ontario hospital (April 1^st^, 2012-March 31^st^, 2019). Poisson regression with robust error variance models were used to estimate the adjusted relative risks of adverse perinatal outcomes for Black people compared with White people while adjusting for covariates.

**Results:**

Among 412,120 eligible pregnant people, 10.1% were Black people and 89.9% were White people. Black people were at an increased risk of gestational diabetes mellitus, preeclampsia, placental abruption, preterm birth (<37, <34, <32 weeks), spontaneous preterm birth, all caesarean sections, emergency caesarean section, low birth weight (<2500g, <1500g), small-for-gestational-age (<10th percentile, <3^rd^ percentile) neonates, 5-minute Apgar score <4 and <7, neonatal intensive care unit admission, and hyperbilirubinemia requiring treatment but had lower risks of elective caesarean section, assisted vaginal delivery, episiotomy, 3rd and 4th degree perineal tears, macrosomia, large-for-gestational-age neonates, and arterial cord pH≤7.1, as compared with White people. No difference in risks of gestational hypertension and placenta previa were observed between Black and White people.

**Conclusion:**

There are differences in several adverse perinatal outcomes between Black and White people within the Ontario health care system. Findings might have potential clinical and health policy implications, although more studies are needed to further understand the mechanisms.

## Introduction

Racial inequities have been recognized as an emergent issue in public health [1, 2]. Maternal race is an important focus of interest for maternal and childbirth research since its relationship with perinatal outcomes have been frequently reported, including stillbirth, preterm birth (PTB), gestational diabetes mellitus (GDM), preeclampsia (PE), and large-for-gestational-age births [3, 4]. Recent studies from the United States (U.S.) showed that non-Hispanic Black pregnant people were at an increased risk for several maternal morbidities, while their neonates had an increased risk of mortality compared with their non-Hispanic White counterparts [5–7]. Similarly, a Canadian study found an increased risk of preterm birth for Black pregnant people compared to White pregnant people [8]. Two other studies (using the same study population) examined live births in Quebec, Canada from 1981–2010 [9, 10]. The results of these two studies showed that compared to Canadian-born pregnant individuals, those originally from Haiti had a higher risk of PTB [adjusted odds ratio (aOR) 1.44, 95% confidence interval (CI) 1.36–1.52], low birth weight (aOR 1.42, 95% CI 1.34–1.50), small-for-gestational-age infants (aOR 1.09, 95% CI 1.05–1.14), and stillbirth rates [adjusted hazard ratio (aHR) 1.76, 95% CI 1.47–2.11) [9, 10].

Researchers have defined race as a social construct [11, 12]. Studies have reported that adverse perinatal outcomes are associated with environmental, biological, and behavioural factors, as well as racial discrimination and socio-economic status (SES) [1, 12, 13]. Increasingly, there is an understanding that racial inequities are not the result of biological or behavioural differences but rather arise from intersectional systems of power and privilege [14]. While the exact mechanism through which these factors interplay is not entirely known, evidence has indicated that racialized pregnant people may be at a greater risk of exposure to chronic stressors in their lifetime, which may augment both psychosocial and physiological factors and heighten the risk of poor perinatal outcomes [15–19]. Discrepancies in access to prenatal care as well as insurance coverage contribute to inconsistent health outcomes in this population [12, 20–22]. A study on perinatal outcomes of uninsured immigrant, refugee, and migrant pregnant individuals in Toronto found 40.4% of Caribbean people to be uninsured—the largest of any other ethnic group sampled [23]. Moreover, the social context experienced by racialized groups exacerbates the effects on their overall health [24, 25]. Characterized by higher rates of poverty and incarceration, Black and other minority groups are potential victims of institutionalized racism, a stressor known to adversely affect health long term [24, 25].

Recently, our research group has published three papers on race using Ontario population data [26–28]. We found that compared with White pregnant people, Asian pregnant people had a higher risk of inadequate gestational weight gain (GWG) and a lower risk of excessive GWG in all weight classes, and non-underweight Black pregnant people had a higher risk of inadequate GWG and a lower risk of excessive GWG [28]. Our findings also showed that excessive GWG contributed more to LGA neonates than pre-pregnancy obesity in White and Asian people [27], while there was no difference between excessive GWG and pre-pregnancy obesity in their contributions to the LGA neonates in Black people [27]. Moreover, our results showed significant differences in the types of adverse perinatal outcomes observed between Asian and White people [26].

Inequities between Black and White pregnant people in perinatal outcomes have been well documented in the U.S., while current Canadian literature seems to lack the same breadth and quantity [17, 29–31]. This discrepancy may be accounted for by the distinct differences in histories, geographic origin, and healthcare system design [31, 32]. In 2016, over 50% of Canada’s Black population were reported to be immigrants [32], compared to only 9% in the U.S. [33]. Indeed, most studies that look at ethnic or racial differences in birth outcomes in Canada report maternal place of origin or place of birth instead of monolithic racial groups like in the U.S [17, 29–31].

A recurring approach found in the aforementioned studies is the use of Caribbean and sub-Saharan African as proxies to refer to Black people in Canada [9, 10, 23]. This approach can potentially exclude native-born individuals who do not identify with a particular country outside of Canada as well as include non-Black people from those regions. Consequently, it is becoming increasingly important to generate more holistic findings to better understand the health outcomes among Black Canadians [8–10, 23]. The Black population currently accounts for 3.5% of Canada’s total population; in Ontario it represents 4.7%, in the Atlantic provinces 1.4%, in Quebec 4%, in the Prairies 2.8%, in British Columbia 1%, and in the Territories 1.2% [31]. Ontario accounts for over 52% of the Black population in Canada, compared to 2.6% in the Atlantic provinces, 26.6% in Quebec, 14.6% in the Prairie provinces, 3.6% in British Columbia, and 0.1% in the Territories, and is thus a well-suited population in which to explore differences in adverse maternal and birth outcomes between Black and White people [32]. As such, we conducted a retrospective population-based cohort study to analyze inequities in adverse perinatal outcomes between Black and White people in Ontario.

## Materials and methods

### Study design and data source

We conducted a population-based retrospective cohort study. Data from the Better Outcomes Registry & Network (BORN) Ontario birth registry was used, which contains comprehensive perinatal information including maternal obstetrical history, environmental and behavioural exposures, and health behaviours and characteristics [34] Data on all hospital deliveries in Ontario are collected in the BORN Information System (BIS). We linked the aggregate pregnancy dataset to the prenatal screening follow-up dataset and aggregate infant dataset from the BIS.

### Study population

We included pregnant people who had a prenatal screening test, and a pregnancy that resulted in a singleton birth (stillbirth and live birth) in any Ontario hospital from April 1^st^, 2012 to March 31^st^, 2019. We excluded pregnant people with missing information on race or who were classified as mixed or a race other than White or Black.

### Exposure and covariates

The exposure variable was maternal race (Black/White), which was self-reported and recorded on prenatal screening requisition forms by care providers. In Ontario, all pregnant people are routinely offered a multiple marker screening test (a conventional prenatal screening test) and can access the test free of charge (personal communication with clinical experts).

We determined the potential confounders based on a literature review and previous research experience. A series of relevant factors that could be potential confounders for the association between maternal race and perinatal outcomes were considered, which included: maternal age at delivery [1, 4, 9, 10, 29, 35, 36], neighborhood household income, parity [1, 9, 10, 30, 35, 36], pre-existing physical health problems (hypertension or diabetes or heart disease or pulmonary disease) [1], mental health condition (a composite measure of depression and anxiety) before or during pregnancy, previous caesarean section [1], pre-pregnancy body mass index (BMI) [defined as height in kilograms (kg) divided by weight in metres squared (m^2^)] [1, 37], assisted reproductive technology (ART) [1], drug use/alcohol exposure/smoking during pregnancy [1, 29, 37, 38], maternal residence area [4, 36], obstetrician in antenatal care team [4, 36, 37], hospital level of maternal care at delivery and infant sex (female, male). Neighbourhood household income and maternal residence area data was derived from the 2016 Canadian census data through the maternal residence postal code [28].

### Outcome measures

We considered a number of primary adverse maternal and neonatal complications and included the following perinatal outcomes: GDM, gestational hypertension, PE, placenta previa, placental abruption, preterm birth (<37, <34, <32 weeks), spontaneous preterm birth, caesarean sections (all, elective, emergency), assisted vaginal delivery, episiotomy, and 3rd and 4th degree perineal tears. Neonatal outcomes included low birth weight (LBW) (<2500 g, <1500 g), macrosomia (>4000 g), small- for-gestational-age (SGA) neonates (defined as <10^th^ percentile of birth weight for gestational age), SGA neonates (<3^rd^ percentile), large-for-gestational-age (LGA) neonates (defined as >90^th^ percentile of birth weight for gestational age), 5-min Apgar score (<7, <4), arterial cord pH ≤7.1, hyperbilirubinemia requiring treatment, and neonatal intensive care unit (NICU) admission. We selected these outcomes based on previous research, data availability, sufficient sample size and group discussion in our research team [26]. These selected outcomes were approved by our internal research protocol review committee and were specified in our protocol for the Research Ethics Board approval. We set values to missing if values of birth weight were outside of the range of 250 g–6000 g and values of arterial cord pH were outside of the range of 6.6–7.4.

### Statistical analysis

Baseline characteristics between Black and White people were compared first. Continuously distributed variables were presented by mean ± standard deviation (SD) and compared using t-tests, and categorical variables were displayed by counts and percentages and compared using chi-square tests.

Afterwards, perinatal outcomes between White and Black people were compared. Poisson regression with robust error variance models were used to estimate the adjusted relative risks (aRR) of adverse perinatal outcomes for Black people compared with White people [39]. Crude relative risk (RR) and aRR with their 95% confidence intervals (CI) were used to estimate the associations between maternal race (Black vs White) and perinatal outcomes. The covariates included in the multivariable models were maternal age at delivery (≤18, 19–24, 25–29, 30–34, 35–39, ≥40 years) [1, 4, 29, 36], neighbourhood household income quintile (lowest, 2nd, 3th, 4th, highest), pre-existing physical health problems (hypertension or diabetes or heart disease or pulmonary disease), pre-existing mental health problems (a composite measure of depression and anxiety) [1, 35], previous caesarean section (yes, no) [1], pre-pregnancy BMI (<18.5, 18.5–24.9, 25.0–29.9, 30–34.9, 35–39.9, ≥40 kg/m^2^), parity (0, ≥1) [1, 30, 36], ART (yes, no) [1], and alcohol exposure or drug use or smoking during pregnancy (yes, no) [1, 29, 37, 38]. For the birth outcomes, we also adjusted for infant sex (female, male) except for the outcomes of small-for-gestational-age neonates (<10th percentile, <3rd percentile) and large-for-gestational-age neonates (>90th percentile), which were calculated after taking infant sex into account. For procedure related maternal outcomes including spontaneous preterm birth, assisted vaginal delivery, episiotomy, all caesarean section, elective caesarean section, emergency caesarean section and 3^rd^ and 4^th^ degree vaginal tears, we further adjusted for maternal residence area (rural, urban), the presence of an obstetrician on the antenatal care team (yes, no), and hospital level of maternal care (I, II, III) at delivery [4, 36, 37, 40]. Covariates were adjusted in multivariable models for each perinatal outcome separately [41, 42].

Multiple imputation methods were used to account for missing covariate data in the regression analysis, in which five datasets were imputed by using fully conditional specification (FCS) logistic regression method [43–45]. Statistical Analysis System (SAS) for Windows, version 9.4 (SAS Institute, Cary, NC) was used to perform the analysis in this study, along with two-tailed tests and a significance level of p<0.05.

### Ethics

This study was approved by the Research Ethics Board at the Children’s Hospital of Eastern Ontario (file number: CHEOREB# 20/15PE). We used only registry and administrative data in this study. In accordance with privacy laws and the Personal Health Information Protection Act in Ontario, Canada, “participant consent” was not required for this research.

## Results

**Fig 1:**
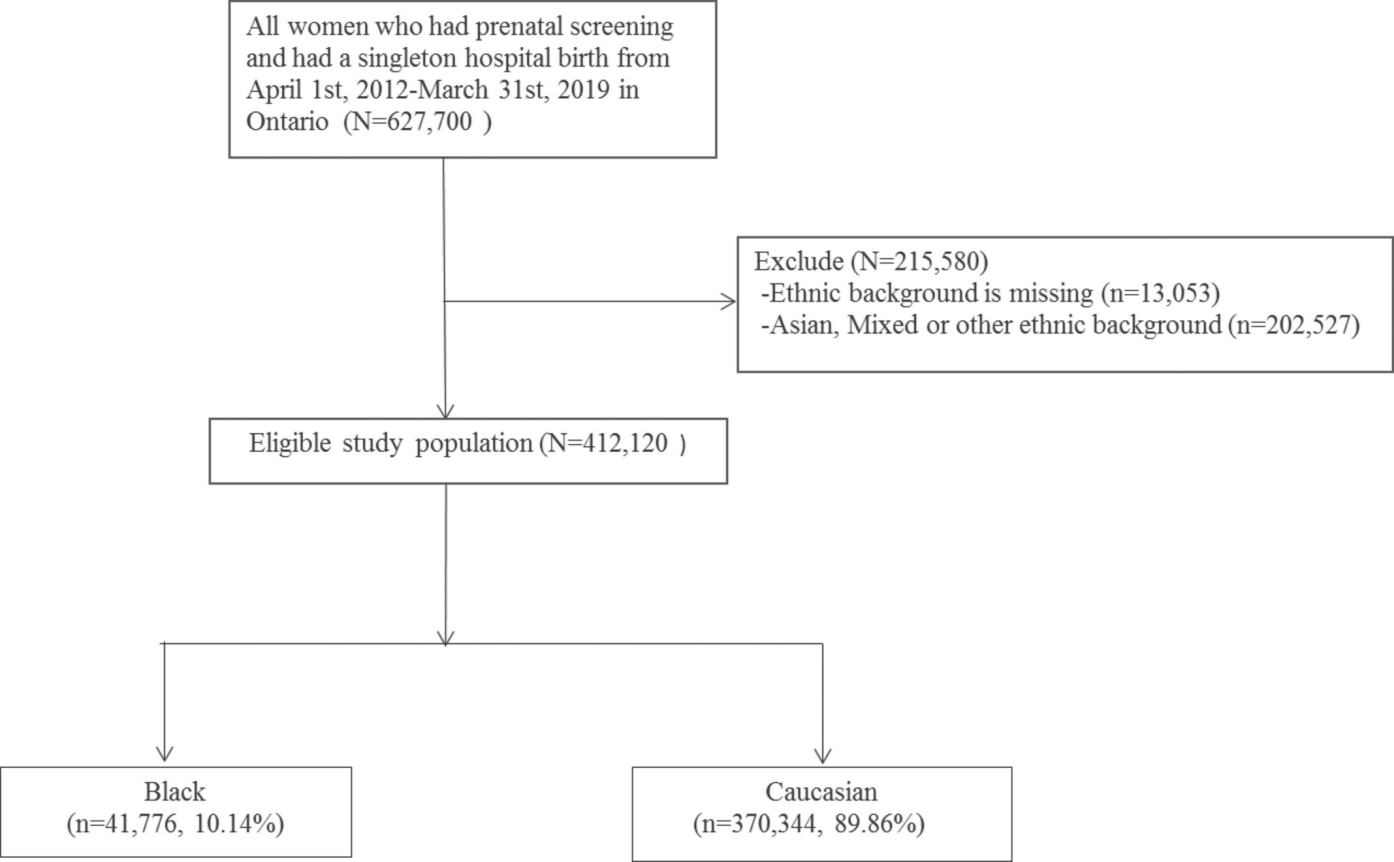
Flow chart of the study cohort and analysis population.

A total of 412,120 eligible pregnant people (10.1% Black people and 89.9% White people) were included in the final analysis. Fig 1 (flow chart of the study cohort and analysis population) shows the number of pregnant people who received prenatal screening and had a singleton hospital birth from April 1^st^, 2012 to March 31^st^, 2019 in Ontario (n = 627,700), as well as the number of pregnant people who were excluded due to missing information (n = 13,053) or a racial background outside of the scope of our investigation such as Asian, mixed, or other (n = 202,527). The resulting eligible study population included 41,776 pregnant people who were Black and 370,344 who were White.

Table 1 shows the distributions of maternal characteristics between Black and White pregnant people. Compared to White pregnant people, Black pregnant people had a higher rate of stillbirths with statistical significance (0.37% of 370,344 White people vs. 0.83% of 41,776 Black people). Black pregnant individuals tended to be younger with an average age (mean ± SD) of 30.82 ± 5.79 compared to 31.03 ± 5.05 for White people. Compared to White people, Black people were more likely to be in the lowest household income quintile (42.97% vs 16.22%), to have a higher BMI (26.94 ± 6.31 vs 25.69 ± 6.18), to have an obstetrician on the antenatal care team (80.81% vs 72.02%), and to be parous (62.09% vs 52.51%). Compared to White people, fewer Black people had a maternal pre-existing health condition (7.27% vs 7.81%), had a mental health condition (6.51% vs 16.82%), had a previous caesarean section (77.66% vs 82.96%), used ART to assist with conception (1.79% vs 4.13%), smoked (3.81% vs 10.25%) or were exposed to drugs (1.46% vs 1.93%) or alcohol (1.58% vs 2.47%) during pregnancy, and delivered in a lower maternal level of care hospital (3.69% vs 11.08%).The majority of both Black and White pregnant individuals lived in urban areas.

**Table 1:** Comparison of characteristics between black and white pregnant people in Ontario, Canada, April 1^st^, 2012-March 31^st^, 2019 (N = 412,120).

Characteristics	Black		White		P value
	n	%	n	%	
	41,776	10.14	370,344	89.86	
Maternal Age at delivery (years) (Mean ± SD)	30.82 ± 5.79		31.03 ± 5.05		< .0001
≤18	489	1.17	3172	0.86	<0.001
19–24	5936	14.21	35787	9.66	
25–29	10471	25.06	94432	25.50	
30–34	13260	31.74	145216	39.21	
35–39	8966	21.46	77190	20.84	
≥40	2602	6.23	14333	3.87	
Missing	52	0.12	214	0.06	
Neighbourhood median household income quintiles (linked to 2016 Canadian Census data)					<0.0001
Quintile 1 (lowest)	17952	42.97	60065	16.22	
Quintile 2	8859	21.21	67886	18.33	
Quintile 3	7408	17.73	75276	20.33	
Quintile 4	4250	10.17	84421	22.80	
Quintile 5 (highest)	2314	5.54	74802	20.20	
Missing	993	2.38	7894	2.13	
Maternal pre-existing health condition					<0.0001
No	38739	92.73	341424	92.19	
Yes	3037	7.27	28920	7.81	
Mental health condition before or during pregnancy					<0.0001
No	35513	85.01	283996	76.68	
Yes	2718	6.51	62309	16.82	
Missing	3545	8.49	24039	6.49	
Previous caesarean section					
Yes	32444	77.66	307232	82.96	<0.0001
No	7229	17.30	49745	13.43	
Missing	2103	5.03	13367	3.61	
Pre-pregnancy BMI (kg/m^2^) (Mean ± SD)	26.94 ± 6.31		25.69 ± 6.18		<0.0001
Underweight (<18.5)	1322	3.16	14371	3.88	<0.0001
Normal (18.5–24.9)	12748	30.52	164173	44.33	
Overweight (25.0–29.9)	9778	23.41	78033	21.07	
Obese (30–34.9)	5204	12.46	36528	9.86	
Obese (35–39.9)	2069	4.95	17008	4.59	
Obese (≥40)	1321	3.16	10792	2.91	
Missing	9334	22.34	49439	13.35	
Parity					<0.0001
0	15281	36.58	172188	46.49	
≥1	25938	62.09	194480	52.51	
Missing	557	1.33	3676	0.99	
Conception by assisted reproductive technology					<0.0001
No	38175	91.38	334407	90.30	
Yes	748	1.79	15278	4.13	
Missing	2853	6.83	20659	5.58	
Drug use during pregnancy					<0.0001
No	38039	91.05	341720	92.27	
Yes	610	1.46	7149	1.93	
Missing	3127	7.49	21475	5.80	
Alcohol exposure during pregnancy					<0.0001
No	38056	91.10	340033	91.82	
Yes	660	1.58	9163	2.47	
Missing	3060	7.32	21148	5.71	
Smoking during pregnancy (any time)					<0.0001
No	37983	90.92	318060	85.88	
Yes	1590	3.81	37972	10.25	
Missing	2203	5.27	14312	3.86	
Pregnancy outcome					<0.0001
Live birth	41430	99.17	368983	99.63	
Stillbirth	346	0.83	1361	0.37	
Infant sex					0.03
Female	20556	49.21	180184	48.65	
Male	21179	50.7	189836	51.26	
Missing or indeterminate	41	0.09	324	0.09	
Maternal residence area					<0.0001
Urban	40720	97.47	312844	84.47	
Rural	364	0.87	51957	14.03	
Missing	692	1.66	5543	1.50	
Obstetrician on antenatal care team					<0.0001
No	8018	19.19	103626	27.98	
Yes	33758	80.81	266718	72.02	
Hospital level of maternal care ^a^					<0.0001
Level I	1543	3.69	41036	11.08	
Level II	30154	72.18	225910	61.00	
Level III	9900	23.70	102145	27.58	
Missing	179	0.43	1253	0.34	

Abbreviation: BMI = body mass index; SD = standard deviation

a. Maternal hospital level of care classification based on newborn and maternal needs, risk and illness as defined by the Provincial Council for Maternal and Child Health (PCMCH) in Ontario.

Table 2 shows the risks of adverse maternal outcomes between Black and White pregnant people. When compared to their White counterparts, Black pregnant people were at an increased risk of GDM (aRR 1.08, 95% CI: 1.04–1.13), PE (aRR 1.1, 95% CI 1.05–1.14), placental abruption (aRR 1.11, 95% CI 1.00–1.22), early preterm birth [<37 weeks (aRR 1.41, 95% CI 1.37–1.44), <34 weeks (aRR 2.29, 95% CI 2.23–2.35), <32 weeks (aRR 2.85, 95% CI 2.78–2.92)], spontaneous preterm birth (aRR 1.24, 95% CI 1.17–1.30), all caesarean section (aRR 1.11, 95% CI 1.09–1.12), and emergency caesarean section (aRR 1.42, 95% CI 1.40–1.44). By contrast, they had lower risks for elective caesarean section (aRR 0.91, 95% CI 0.89–0.93), assisted vaginal delivery (aRR 0.83, 95% CI 0.79–0.87), episiotomy (aRR 0.71, 95% CI 0.67–0.76), and 3rd and 4th degree perineal tears (aRR 0.72, 95% CI 0.64–0.81). No difference was observed between Black and White people for risk of gestational hypertension (aRR 1.01, 95% CI 0.96–1.06) or placenta previa (aRR 1.02, 95% CI 0.89–1.15).

**Table 2:** Comparison of risks of adverse maternal outcomes between black and white pregnant people in Ontario, Canada, April 1st, 2012-March 31st, 2019.

Maternal Outcomes ^a^	Black		White (Reference group)		Crude RR (95% CI)	Adjusted RR ^b^^,^^c^^,^^d^ (95% CI)
	n	%	n	%		
Gestational diabetes mellitus	2655	6.36	18811	5.08	1.25 (1.20, 1.30)	1.08 (1.04, 1.13)
Gestational hypertension	1680	4.02	14588	3.94	1.02 (0.97, 1.07)	1.01 (0.96, 1.06)
Preeclampsia	1805	4.32	15032	4.06	1.06 (1.01, 1.12)	1.10 (1.05, 1.14)
Placenta previa	271	0.65	2496	0.67	0.96 (0.85, 1.09)	1.02 (0.89, 1.15)
Placental abruption	365	0.87	3011	0.81	1.07 (0.96, 1.20)	1.11 (1.00, 1.22)
Preterm birth (<37 weeks)	3701	8.86	23500	6.35	1.40 (1.35, 1.44)	1.41 (1.37, 1.44)
Preterm birth (<34 weeks)	1495	3.58	6024	1.63	2.20 (2.08, 2.33)	2.29 (2.23, 2.35)
Preterm birth (<32 weeks)	1108	2.65	3659	0.99	2.68 (2.51, 2.87)	2.85 (2.78, 2.92)
Spontaneous preterm birth	1250	2.99	9021	2.44	1.23 (1.16, 1.30)	1.24 (1.17, 1.30)
All caesarean sections	13764	32.95	105311	28.44	1.26 (1.23, 1.29)	1.11 (1.09, 1.12)
Elective caesarean section	6923	16.57	57075	15.41	0.91 (0.89, 0.94)	0.91 (0.89, 0.93)
Emergency caesarean section	6838	16.37	48231	13.02	1.22 (1.20, 1.25)	1.42 (1.40, 1.44)
Assisted vaginal delivery	2641	6.32	33279	8.99	0.70 (0.68, 0.73)	0.83 (0.79, 0.87)
Episiotomy	2378	5.69	33402	9.02	0.63 (0.61, 0.66)	0.71 (0.67, 0.76)
3^rd^ and 4^th^ degree perineal tears	574	2.15	8697	3.39	0.60 (0.55, 0.65)	0.72 (0.64, 0.81)

Abbreviations: RR = relative risk; CI = confidence interval

^a^ N = 412,120 for all outcomes except for the outcomes of 3^rd^ and 4^th^ degree perineal tears (N = 368118) and elective or emergency caesarean sections (N = 412089) after missing values were removed.

^b^ Multiple imputation methods were used to account for missing covariate data in the regression analysis, in which five datasets were imputed by using fully conditional specification logistic regression method.

^c^ Modified Poisson regression models were used to estimate the relative risks of the outcomes.

^d^ Models were adjusted for maternal age at delivery, neighbourhood household income quintile, previous caesarean section, pre-pregnancy BMI, parity, ART, alcohol exposure or drug use or smoking during pregnancy, pre-existing physical health problems and mental health condition before or during pregnancy. In addition to the aforementioned covariates, we further adjusted for maternal residence area, the presence of an obstetrician on the antenatal care team, and hospital level of maternal care at delivery for outcomes of spontaneous preterm birth, assisted vaginal delivery, episiotomy, all caesarean sections, elective caesarean section, emergency caesarean section and 3rd and 4th degree vaginal tears.

Table 3 shows risks of adverse neonatal outcomes between Black and White pregnant people. Compared to White people, Black people were at a higher risk of LBW (<2500g (aRR 1.96, 95% CI 1.92–2.00), <1500g (aRR 3.03, 95% CI 2.95–3.11), SGA (<10th percentile (aRR 1.77, 95% CI 1.74–1.80), <3^rd^ percentile (aRR 2.09, 95% CI 2.03–2.15), 5 min Apgar score <4 (aRR 2.29, 95% CI 2.20–2.38) and <7 (aRR 1.63, 95% CI 1.57–1.69), NICU admission (aRR 1.25, 95% CI 1.22–1.27), and hyperbilirubinemia requiring treatment (aRR 1.21, 95% CI 1.19–1.24). Comparably, Black pregnant people were at lower risk for macrosomia (aRR 0.61, 95% CI 0.57–0.64), LGA (aRR 0.64, 95% CI 0.60–0.67), and arterial cord pH≤7.1 (aRR 0.93, 95% CI 0.88–0.98) compared to White people.

**Table 3:** Comparison of risks of adverse neonatal outcomes between black and white pregnant people in Ontario, Canada, April 1st, 2012-March 31st, 2019.

Birth outcome ^a^	Black		White (Reference group)		Crude RR (95% CI)	Adjusted RR ^b^^,^^c^^,^^d^ (95% CI)
	n	%	n	%		
Low birth weight (<2500g) (N = 409644)	3332	8.03	16059	4.36	1.84 (1.78, 1.91)	1.96 (1.92, 2.00)
Low birth weight (<1500g) (N = 409644)	989	2.38	2896	0.79	3.03 (2.82, 3.26)	3.03 (2.95, 3.11)
Macrosomia (>4000g) (N = 409644)	3273	7.89	44301	12.03	0.66 (0.63, 0.68)	0.61 (0.57, 0.64)
SGA (<10^th^ percentile) (N = 406909)	5001	12.16	27290	7.46	1.63 (1.58, 1.68)	1.77 (1.74, 1.80)
SGA (<3^rd^ percentile) (N = 406909)	1423	3.46	6654	1.82	1.90 (1.80, 2.01)	2.09 (2.03, 2.15)
LGA (>90^th^ percentile) (N = 406909)	3243	7.88	40867	11.17	0.71 (0.68, 0.73)	0.64 (0.60, 0.67)
5-minute Apgar score <7 (N = 407702)	1454	3.53	8542	2.33	1.51 (1.43, 1.60)	1.63 (1.57, 1.69)
5-minute Apgar score <4 (N = 407702)	668	1.62	2631	0.72	2.26 (2.08, 2.46)	2.29 (2.20, 2.38)
Arterial cord pH ≤7.1 (N = 352833)	1827	5.15	18688	5.89	0.87 (0.83, 0.92)	0.93 (0.88, 0.98)
NICU admission (N = 412120)	5904	14.13	43149	11.65	1.21 (1.18, 1.24)	1.25 (1.22, 1.27)
Hyperbilirubinemia requiring treatment (N = 412120)	6005	14.37	44761	12.09	1.19 (1.16, 1.22)	1.21 (1.19, 1.24)

Abbreviations: RR = relative risk; CI = confidence interval; NICU = newborn intensive care unit; SGA = Small-for-gestational-age neonates

LGA = Large-for-gestational-age neonates

^a^ Missing values were removed from outcomes.

^b^ Multiple imputation methods were used to account for missing covariate data in the regression analysis, in which five datasets were imputed by using fully conditional specification logistic regression method.

^c^ Modified Poisson regression models were used to estimate the relative risks of the outcomes.

^d^ Covariates included maternal age at delivery, neighbourhood household income quintile, previous caesarean section, pre-pregnancy BMI, parity, ART, alcohol exposure or drug use or smoking during pregnancy, pre-existing physical health problems and mental health condition before or during pregnancy for outcomes of small-for-gestational-age neonates (<10^th^ percentile, <3^rd^ percentile) and large-for-gestational-age neonates (>90th percentile). In addition to the aforementioned covariates, we further adjusted for infant sex for outcomes of low birth weight (<2500g, (<1500g), macrosomia, 5-minute Apgar score <4 and <7, arterial cord pH ≤7.1, NICU admission and hyperbilirubinemia requiring treatment.

## Discussion

### Main findings

In this population-based study, we found that compared with White pregnant people, Black pregnant people had higher rates of stillbirths and were likely at an increased risk of several poor maternal and neonatal outcomes, including GDM, PE, placental abruption, preterm birth, emergency caesarean section, LBW, SGA, 5-minute Apgar score <4 and <7, NICU admission, and hyperbilirubinemia requiring treatment. Conversely, even after adjustment for social and economic factors, Black pregnant people had lower risks of elective caesarean section, assisted vaginal delivery, episiotomy, 3rd and 4th degree perineal tears, macrosomia, LGA, and arterial cord pH≤7.1, compared with White people. No difference between Black and White people was observed for risk of gestational hypertension or placenta previa.

### Interpretations

Ontario has universal coverage for physician and hospital services, so access to healthcare services should theoretically not be a barrier. However, we found differences in perinatal outcomes between White and Black people. In this study, the greatest observed differences in adverse maternal outcomes between Black and White pregnant people were very preterm birth (<32 weeks), followed by moderate preterm birth (<34 weeks). Our findings that Black pregnant people have an increased risk of preterm birth is consistent with other literature [8, 46–50]. A recent systematic review found that Black pregnant people had a 51.1% higher risk of preterm birth compared to non-Black pregnant people (RR 1.51, 95% CI 1.39–1.65), which is similar to our study results (RR: 1.41, 95% CI 1.37–1.44) for preterm birth (<37 weeks) [51]. One comparative study on Black-White inequities in preterm birth between Canada and the U.S. also found a similar magnitude with adjusted RR of 1.46 (95% CI 1.29–1.63) and 1.41 (95% CI 1.40–1.42), respectively, with White pregnant people as a reference group [8].

Another important difference in adverse maternal outcomes was that Black pregnant people had higher rates of all caesarean sections and emergency caesarean sections, which is supported by previous studies [52, 53]. Our findings may be explained by Black pregnant people’s increased likelihood of non-reassuring fetal status and by systematic discrimination in delivery type [52, 54]. Moreover, Black pregnant people in this study were also found to be at higher risk for GDM, PE, and placental abruption, which is supported by other studies [55–57]. As GDM is associated with an increased risk of PE, this result is unsurprising due to higher risk of obesity among Black pregnant people. Black people may be more likely to suffer from unhealthy and inactive lifestyles, but this may be a result of systemic racism rather than of their own choosing, which increases the risk of GDM [58].

In our study, Black pregnant people were less likely to have 3^rd^ and 4^th^ degree perineal tears. One potential explanation for this finding may be that Black pregnant women are less likely to have assisted vaginal deliveries, which are associated with increased rates of 3rd and 4th degree lacerations [59]. Our results also showed that Black pregnant people were less likely to develop macrosomia and be nulliparous, both of which may be protective against perineal tears [60]. Another significant finding was that Black pregnant people were less likely to receive an episiotomy and assisted vaginal delivery, which is supported by previous research [61]. Although the actual mechanism is unknown, this finding may be partially explained by potential negative differential treatment towards racialized minorities [54, 62]. Further studies are needed to evaluate if health care providers are less likely to offer Black pregnant people assisted vaginal deliveries, which decreases the risk of 3rd and 4th degree lacerations and increases the risk of emergency caesarean sections for Black pregnant people.

In terms of neonatal outcomes, the most substantial difference was very low birth weight (VLBW, <1500g). Compared to White pregnant people, Black pregnant people had an increased risk of giving birth to VLBW babies by 200%. In addition, the risk of very small neonates (<3^rd^ percentile) and low 5-minute Apgar scores (<4) was increased by 100% among Black pregnant people. These findings are consistent with other research in the U.S, which does not have universal health care, and England, which has universal health care [63–68]. These racial inequities have in part been explained by the weathering hypothesis, which points to chronic exposure to social and economic disadvantage as a major factor in causing adverse outcomes among this group [69]. Although the literature does suggest potential polymorphisms as a cause for preterm birth, this finding does not seem to hold well in a context where Black pregnant people face a significant number of stressors on account of racial and related discrimination [70].

SGA seems to be a common adverse outcome among Black pregnant people, which has been linked to obesity and vitamin D deficiency [71–73]. Black pregnant people’s higher risk of obesity can be attributed to higher prevalence of food insecurity and more positive cultural attitudes towards bigger pregnant people [74]. With an increased risk of many adverse maternal and neonatal outcomes, it is unsurprising that the infants of Black pregnant people also experience higher rates of NICU admission. Indeed, studies show that characteristics of such neonates included LBW, low gestational age, preterm birth, and delivery by caesarean section, with multiple births being a defining maternal characteristic [75]. Consequently, higher rates of neonatal mortality were also found among this population, with one study finding a HR of 1.38 (95% CI 1.01–1.89) for non-Spanish Caribbean pregnant individuals, 1.32 (95% CI 1.05–1.66) for sub-Saharan African pregnant individuals, and 2.29 (95% CI 1.90–2.76) for Haitian pregnant individuals when compared to their Canadian-born counterparts [76]. Other findings in our study included Black pregnant people being at lower risk of LGA and low arterial cord PH. These observations align with other research that showed large for gestational age to be correlated with lower umbilical cord pH [77]. Interestingly, the literature has established a link between pre-pregnancy obesity and LGA neonates [27], yet this phenomenon was not observed in this study, with Black pregnant people experiencing a lower risk for LGA despite being at a higher risk for obesity. Reasons for this finding include the presence of multiple other adverse outcomes such as preterm birth and LBW that favoured the development of SGA neonates. Indeed, studies show increased risk for SGA when placental abruption, PE, and gestational hypertension are present [78].

Although Ontario has a universal healthcare system, this does not mean that all pregnant people experience the same level of trust and respect from providers, nor does it mean that all pregnant people have equal access to care, with some facing barriers related to transportation, childcare, and equal access to living wages. As demonstrated in this study, despite a universal healthcare system, racial disparities in maternal and newborn outcomes still exist, suggesting there may be other factors related to the social determinants of health. One systematic review study examined 15 health equity studies and found that health care providers’ attitudes to White people and to people of color are different [79]. The researchers reported that implicit bias (unconscious bias) among health care professionals may affect patients’ health outcomes [79]. Furthermore, one recent study conducted in Toronto, Ontario, Canada confirmed that there is healthcare provider bias in the Canadian healthcare system [80]. Interestingly, in our study, we found that Black pregnant people were more likely to have an obstetrician on an antenatal care team compared to their White counterparts but were more likely to experience several adverse outcomes of interest. This finding also suggests the potential existence of maternal healthcare provider bias. While more research is needed to evaluate this bias further, the Ontario healthcare system needs interventions to address healthcare provider bias since it may affect relationships between patients and healthcare providers and negatively impact patient outcomes [79, 80]. Moreover, additional ancillary support might be required for at risk minority groups.

As a next step, we plan to present our data to the Black community of Ontario. In addition, future research should be more transparent and include collaboration with community members in all phases of the research. Finally, intersectional approaches are growing, where researchers look at outcomes within race across categories such as age, income, education, and marital status [81]. We plan to adopt an intersectional approach in our future studies.

### Strengths and limitations

There are several strengths of this study. Firstly, this study is based on a large population with comprehensive demographic and health care information, allowing an investigation of many adverse perinatal outcomes with appropriate adjustment for potential confounding factors. Secondly, this study is the first of its kind to study perinatal inequities among Black pregnant people in Ontario. Previous studies were either limited to other provinces, specific cities, or the country as a whole [9, 10, 23, 46, 76, 82–84]. Third, universal access to quality maternity care helped to isolate maternal factors from health care factors.

Limitations of this study should be acknowledged. Firstly, in this study the terms Black and White pregnant people included people who may be from different religions, cultures, immigrant statuses, and other demographic characteristics. Black and White pregnant people from different backgrounds may be at different risk regarding maternal and birth outcomes. However, due to data limitations, we were not able to study the effects of different demographic factors on this study population, which may have limited the specificity of our results. Secondly, self-reported race may result in some misclassifications, either by including individuals who fall outside of our intended target group or by excluding individuals who fall within it. For example, while the form is intended to solicit self-reported race, there is no evidence to indicate how often this question is completed at the sole discretion of the provider. For example, the care provider who orders the prenatal screening test may select the maternal ancestry picklist value on the test requisition based on an assumption. Thirdly, all pregnant people are routinely offered free access to conventional multiple marker screening (a regular prenatal screening program) in Ontario. However, only approximately 70% of pregnant people in Ontario receive prenatal screening [85]. Prenatal screening uptake is higher in pregnant people who live urban areas and high-income neighbourhoods; this implies that our study population may represent a more advantaged group than the total population. In addition, it suggests there might be potential barriers for disadvantaged people to access care in this universal healthcare system in Ontario [86]. Future research is needed to examine the perinatal outcomes among all pregnant people in Ontario. Fourthly, in this study, our data was limited to pregnant people in Ontario. While Canadian provinces and territories have similar health care systems, and our study results may be generalizable to other areas of Canada, future research is needed to examine adverse perinatal outcomes in other geographical areas. Finally, literature has suggested that shoulder dystocia may cause severe maternal morbidity and poor neonatal outcomes [87]. We will examine racial variations on this outcome in our future studies.

## Conclusions

In summary, our population-based study found that compared with White pregnant people, Black pregnant people had a higher rate of stillbirths and were at an increased risk of many poor perinatal outcomes including several poor maternal and neonatal outcomes, including GDM, PE, placental abruption, preterm birth, emergency caesarean section, LBW, SGA, 5 min Apgar score <4 and <7, NICU admission, and hyperbilirubinemia requiring treatment but were at a lower risk of elective caesarean section, assisted vaginal delivery, episiotomy, 3rd and 4th degree perineal tears, macrosomia, LGA, and arterial cord pH≤7.1. On the other hand, there was no observed difference between the study groups for risk of gestational hypertension or placenta previa. Given the heterogeneity in the demographic and social characteristics among different Black communities, there is a need for improved measurement of race and ethnicity that elucidates the underlying mechanisms of inequities, such as systemic racism, including implicit bias in health care.

In conclusion, there are differences in several adverse perinatal outcomes between Black and White people within the Ontario health care system. Findings might have potential clinical and health policy implications, although more studies are needed to further understand the mechanisms.

## Abbreviations

aRR: Adjusted Relative Risk
ART: Assisted Reproductive Technology
BIS: BORN Information System
BMI: Body Mass Index
BORN: Better Outcomes Registry & Network
CI: Confidence Interval
FCS: Fully Conditional Specification
GDM: Gestational Diabetes Mellitus
GWG: Gestational Weight Gain
ICU: Intensive Care Unit
LBW: Low Birth Weight
LGA: Large-for-Gestational-Age
NICU: Neonatal Intensive Care Unit
OR: Odds Ratio
PE: Preeclampsia
PTB: Preterm Birth
RR: Relative Risk
SAS: Statistical Analysis System
SGA: Small-for-Gestational-Age
SD: Standard Deviation
SES: Socio-Economic Status
U.S.: United States
VLBW: Very Low Birth Weight
